# MiR-30e inhibits tumor growth and chemoresistance via targeting IRS1 in Breast Cancer

**DOI:** 10.1038/s41598-017-16175-x

**Published:** 2017-11-21

**Authors:** Min-min Liu, Zhi Li, Xue-dong Han, Jian-hua Shi, Dao-yuan Tu, Wei Song, Jian Zhang, Xiao-lan Qiu, Yi Ren, Lin-lin Zhen

**Affiliations:** Department of Breast and Thyroid Surgery, Huai’an First People’s Hospital, Nanjing Medical University, Huai’an, China

## Abstract

MicroRNA-30e (miR-30e) is downregulated in various tumor types. However, its mechanism in inhibiting tumor growth of breast cancer remains to be elucidated. In this study, we found that miR-30e was significantly downregulated in tumor tissues of breast cancer (BC) patients and cell lines, and overexpression of miR-30e inhibited cell proliferation, migration and invasion. To understand the potential mechanism of miR-30e in inhibiting tumor growth, we showed that miR-30e blocked the activation of AKT and ERK1/2 pathways, and the expression of HIF-1α and VEGF via directly targeting IRS1. Moreover, miR-30e regulates cell proliferation, migration, invasion and increases chemosensitivity of MDA-MB-231 cells to paclitaxel by inhibiting its target IRS1. MiR-30e also inhibited tumor growth and suppressed expression of IRS1, AKT, ERK1/2 and HIF-1α in mouse xenograft tumors. To test the clinical relevance of these results, we used 40 pairs of BC tissues and adjacent normal tissues, analyzed the levels of miR-30e and IRS1 expression in these tissues, and found that miR-30e levels were significantly inversely correlated with IRS1 levels in these BC tissues, suggesting the important implication of our findings in translational application for BC diagnostics and treatment in the future.

## Introduction

Breast cancer (BC) is the most common malignancy in women in the world. The mortality of breast cancer over the last several decades has decreased, because of a combination of mammographic screening and improvements in systemic therapy^1^. Neoadjuvant systemic treatment before surgery for advanced breast cancer is considered one of the most crucial factors in reducing mortality^2–5^. Invasion and metastasis remain the main obstacles in the treatment of breast cancer. Thus, research on the molecular mechanisms of breast cancer is receiving increased interest.

MicroRNAs (miRNAs) are 20–22-nucleotide non-coding RNA molecules that negatively regulate gene expression by binding to the 3′-untranslated region (UTR) of their target genes with partial complementarity, leading to degradation of the target mRNAs, inhibition of their translation or both^6,7^. It has been found that miRNAs regulate various pathological behaviors of cancer cells, such as cell proliferation, motility and sensitivity to chemotherapy^8–14^.

The miR-30 family members include miR-30a, miR-30b, miR-30c, miR-30d and miR-30e. The miR-30 family is associated with cell differentiation, cellular senescence, apoptosis, and involved in the pathogenesis of tumors and other disorders of the nervous, genital, circulatory, alimentary and respiratory systems^15–17^. Previous studies reported the downregulation of miR-30 family members during osteoblast differentiation from mouse preosteoblast cell lines^18,19^. miR-30a/b/c/d were demonstrated to be able to negatively regulate BMP-2-induced osteoblast differentiation by targeting Smad1^19^. In contrast, miR-30 family members were upregulated during adipogenic differentiation of adipose tissue-derived stem cells, and miR-30a and miR-30d contributed to adipocyte formation^20^. To date, some genes have been identified as target genes of miR-30e, including Ubc9, Bmi1, P4HA1, ABL and ATG5^21–25^. Furthermore, our present work provides novel evidences which demonstrating that miR-30e inhibits tumor growth and chemoresistance *via* targeting IRS1 in breast cancer.

In this study, we demonstrated that miR-30e levels were downregulated in human breast cancer specimens using 40 pairs of normal and cancer tissues. Then, we will investigate: (1) what is the role of miR-30e in breast cancer cell growth, migration and invasion; (2) what is the direct target of miR-30e that is associated with cancer development; and (3) whether forced miR-30e expression inhibits cell growth, migration, invasion and chemoresistance *via* this direct target. These results will provide new insights into the molecular mechanism of breast cancer as well as provide potential new therapeutic strategy for breast cancer treatment in the future.

## Results

### MiR-30e expression is downregulated in breast cancer tissues and cell lines

**Figure 1:**
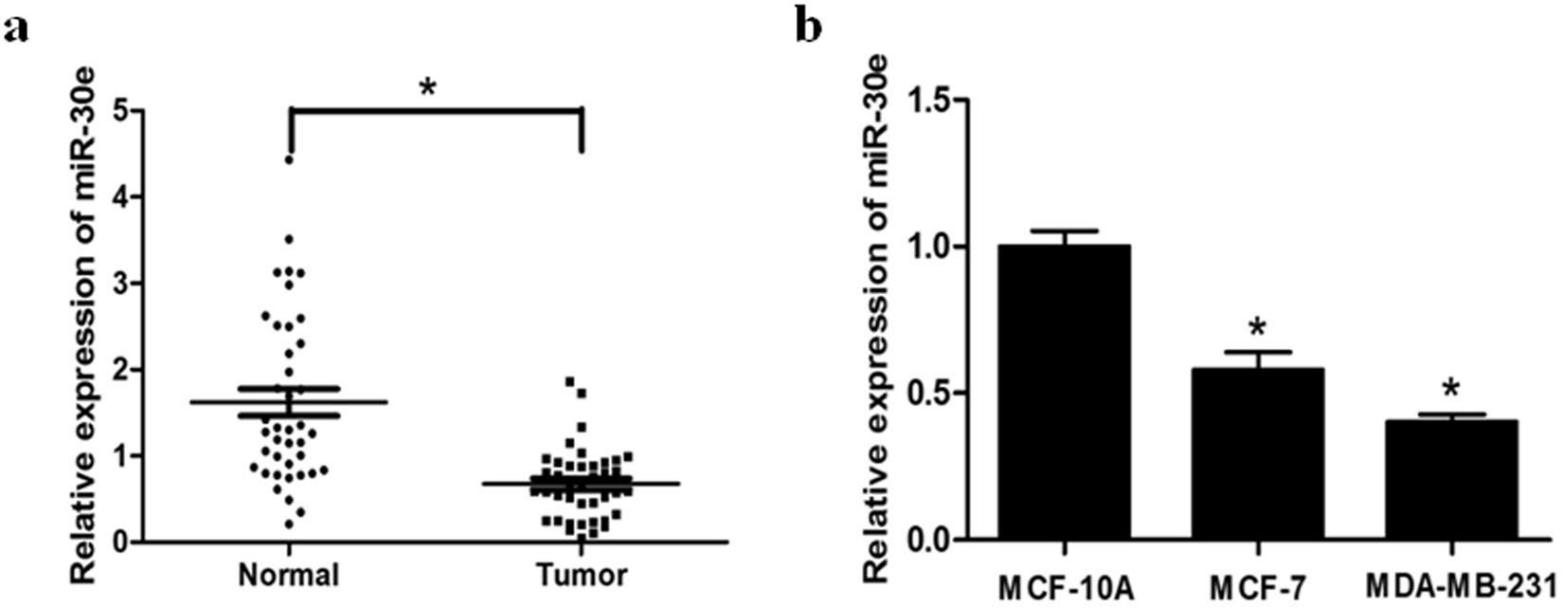
MiR-30e expression is downregulated in breast cancer tissues and cell lines. (**a**) Relative miR-30e expression levels were analyzed by qRT-PCR in 40 paired breast cancer (BC) tissues compared with adjacent non-cancerous tissues. U6 RNA level was used as an internal control. (**b**) Relative miR-30e expression was analyzed in mammary epithelia cell (MCF10A) and BC cell lines, MCF-7 and MDA-MB-231(MDA-MB-231). Data represent mean ± SD. of 3 replicates. *Indicated significant difference at *P* < 0.05.

To evaluate the role of miR-30e in breast cancer, we first investigated the expression levels of miR-30e in normal tissues and breast cancer tissues by qRT-PCR (Fig. 1a). The results showed that the expression of miR-30e was consistently lower in the breast cancer tissues compared with normal tissues. In addition, expression of miR-30e in two breast cancer cell lines MCF-7 and MDA-MB-231, was significantly decreased compared with the normal cells MCF10A (Fig. 1b). Thus, our results indicated that miR-30e was downregulated in breast cancer tissues and cell lines.

### Overexpression of miR-30e inhibits the ability of cell proliferation, cell migration and invasion in BC cells

**Figure 2:**
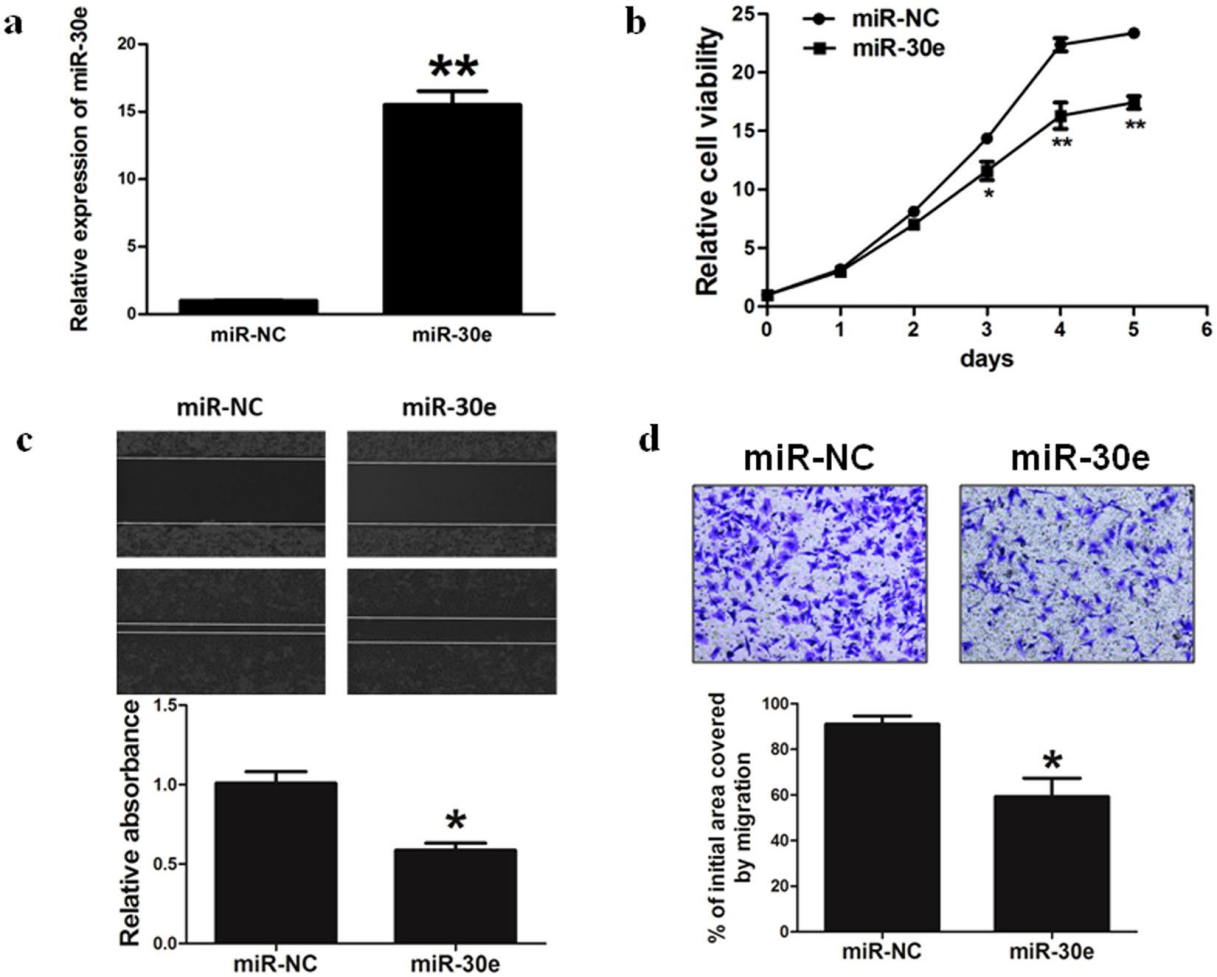
Overexpression of miR-30e inhibits the ability of cell proliferation, migration and invasion in BC cells. (**a**) Relative miR-30e expression levels were analyzed by qRT-PCR in MDA-MB-231/ miR-NC and MDA-MB-231/miR-30e stable cell lines. (**b**) Over-expression of miR-30e arrested cell proliferation in MDA-MB-231 cells. (**c**) Cells were treated as above. A sterile 20 μL pipette tip was used form a wound gap. The wound gaps were photographed (top) and measured (bottom). Forced expression of miR-30e also markedly reduced the wound-healing rate. (**d**) MiR-30e over-expression reduced cell invasion in MDA-MB-231 cells. Data represent mean ± SD. of three replicates. *Indicates significant difference at *P* < 0.05; **indicates significant difference at *P* < 0.01.

To study the role of miR-30e in cancer carcinogenesis, stable cell lines were established (Fig. 2a). miR-30e-overexpressed MDA-MB-231 cells were used to analyze activity of cell growth. The results showed that activity of cell growth were attenuated in miR-30e-overexpressed cells compared with cells expressing miR-NC (Fig. 2b).

Since cell migration and invasion are key characteristics of malignant tumor, next, we investigated the effects of miR-30e on migration and invasion. Over-expression of miR-30e dramatically inhibited the normally strong migration and invasive capacity of breast cancer cells (Fig. 2c,d). Our results suggested that miR-30e-overexpressed suppressed breast cancer cell proliferation, cell migration and invasion.

### MiR-30e inhibits AKT and ERK1/2 pathways via targeting IRS1

**Figure 3:**
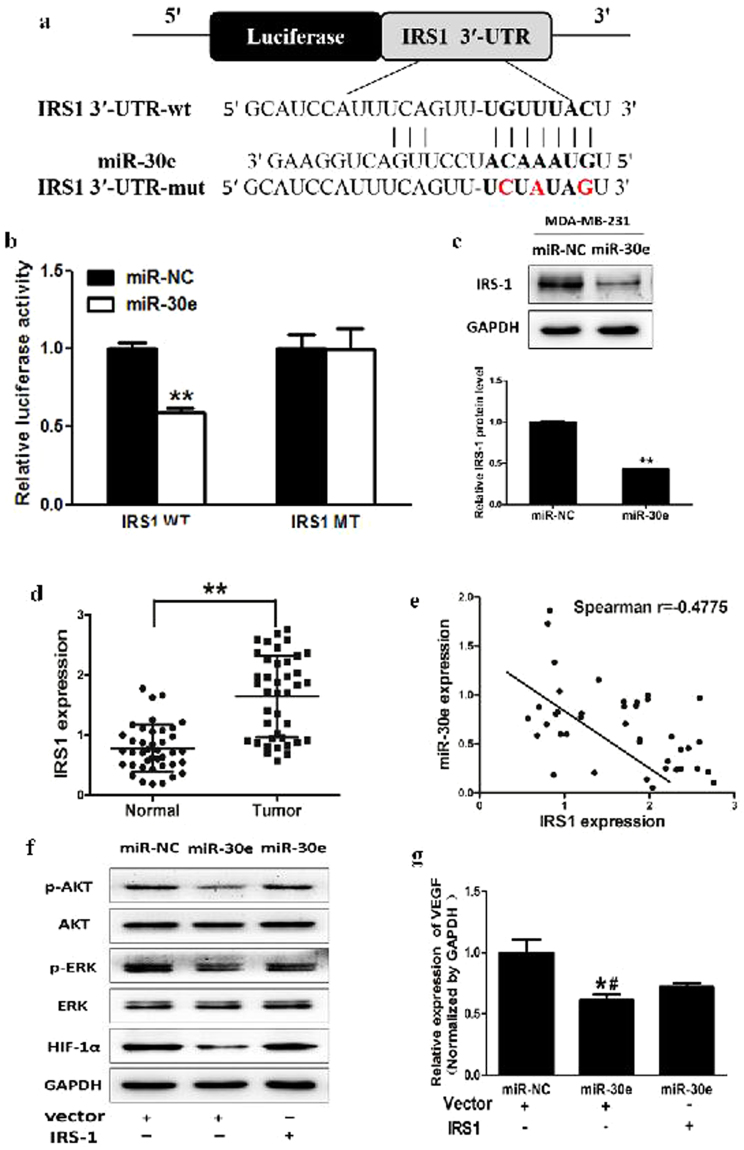
MiR-30e inhibits AKT and ERK1/2 pathways via targeting IRS1. (**a**) Sequence of the miR-30e binding site within the human IRS1 3′-UTR and a schematic diagram of the reporter construct showing the entire IRS1 3′-UTR sequence and the mutant IRS1 3′-UTR sequence. The mutant nucleotides of the IRS1 3′-UTR are labeled in red. (**b**) Luciferase assay on MDA-MB-231 cells, which were co-transfected with miR-NC or miR-30e and a luciferase reporter containing the full length of IRS1 3′-UTR (WT) or a mutant (MT) harboring four mutant nucleotides of the miR-30e binding site. Luciferase activities were measured 24 h post-transfection. MiR-30e markedly suppressed luciferase activity in IRS1 3′-UTR (WT) reporter constructs. The data respresent means ± SEM. for separate transfections (n = 4). (**c**) The immunoblotting showed that expression levels of IRS1 were decreased in cells with miR-30e over-expression. (**d**) The expression levels of IRS1 in normal tissues and human BC specimens were determined by qRT-PCR analysis and fold changes were obtained by the ratios of IRS1 to GAPDH levels. (**e**) Spearman’s correlation analysis was used to determine the correlations between the expression levels of IRS1 and miR-30e in human BC specimens. (**f**) The expression levels of phosphorylated AKT (p-AKT) and phosphorylated ERK1/2 (p-ERK1/2) were decreased in cells with miR-30e over-expression, while AKT and ERK1/2 protein levels remained unchanged. Over-expression of IRS1 restored miR-30e-inhibited cellular protein levels of p-AKT and p-ERK1/2 and HIF-1α. (**g**) Overexpression of IRS1 rescued VEGF mRNA expression inhibited by miR-30e. The VEGF mRNA level was normalized to that of GAPDH. Data represent mean ± SD. of three replicates. **Indicates significant difference at *P* < 0.01. * or ^#^ indicated significant difference at *P* < 0.05. *Indicates significant difference compared to control; ^#^indicates significant difference compared to miR-30e plus IRS1treatment.

To analyze the underlying molecular mechanism of miR-30e in BC, TargetScan and miRanda (www.targetscan.org; www.microrna.org) were used to explore potential targets of miR-30e in BC. Figure 3a shows that the 3′-UTR of IRS1 contained the binding site for the seed region of miR-30e. To confirm that IRS1 was the direct target of miR-30e in BC, human IRS1 3′-UTR, containing either the wild-type or mutant miR-30e binding sequence, was cloned downstream of the firefly luciferase reporter gene in the pMIR-REPORTER vector. MDA-MB-231 cells were transfected with either of the two reporter plasmids, plus either miR-30e or NC vector. The luciferase activity of the reporter in the vector containing the IRS1 3′-UTR WT was significantly reduced by miR-30e, while the IRS1 3′-UTR MT exhibited an insignificantly affected luciferase activity (Fig. 3b). Western blotting were conducted to determine the IRS1 expression at the protein level. We found that the IRS1 expression at the protein level was down-regulated in miR-30e treated cells (Fig. 3c). These results suggested that miR-30e directly targeted IRS1 by binding to its 3′-UTRs in BC cells. Furthermore, we measured levels of IRS1 in human BC specimens and adjacent normal tissues. The results showed that the average expression levels of IRS1 were significantly higher in tumor tissues than those in the adjacent normal tissues (Fig. 3d). Next, we determined the correlation between IRS1 and miR-30e expression levels in the same human BC specimens using Spearman’s rank correlation analysis. As shown in Fig. 3e, expression of IRS1 and miR-30e were inversely correlated in 40 human BC specimens (Spearman’s correlation r = −0.4775).

The PI3K/AKT and MAPK/ERK pathways act as major downstream of IRS1 signaling, which are critical in mitogenesis and oncogenesis, and several downstream factors such as hypoxia-inducible factor-1α (HIF-1α) and vascular endothelial growth factor (VEGF) have been linked to the PI3K/AKT and MAPK/ERK pathways. Cellular levels of p-AKT and p-ERK1/2 were significantly decreased in cells stably expressing miR-30e compared with miR-NC, while no statistically significant reduction of AKT and ERK1/2 were detected (Fig. 3f). Here, we observed that HIF-1α and VEGF levels in miR-30e-expressed cells were both reduced (Fig. 3g). Overexpression of IRS1 restored miR-30e-inhibited protein levels of p-AKT, p-ERK1/2, HIF-1α and VEGF. These results suggested that miR-30e inhibited PI3K/AKT and MAPK/ERK pathways *via* targeting IRS1.

### MiR-30e regulates cell proliferation, migration, invasion and increases chemosensitivity of MDA-MB-231 cells to paclitaxel by inhibiting its target IRS1

**Figure 4:**
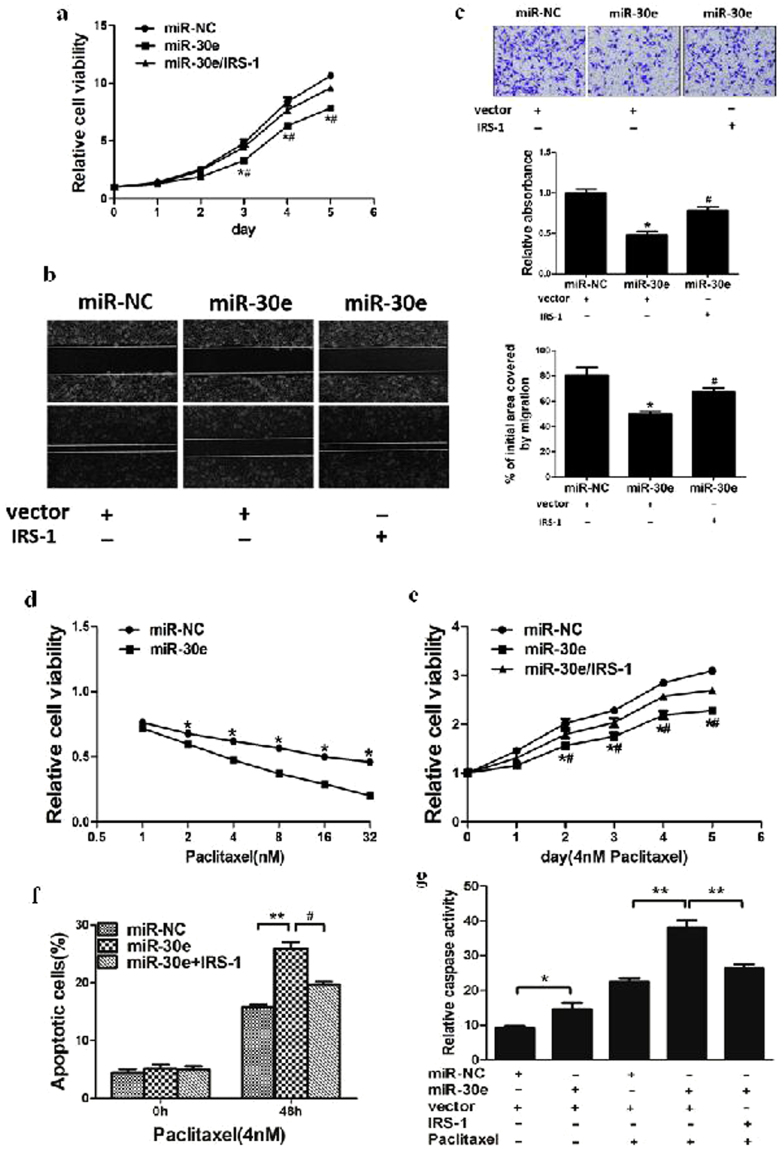
MiR-30e regulates cell proliferation, migration, invasion and increases chemosensitivity of MDA-MB-231 cells to paclitaxel by inhibiting its target IRS1. (**a**) Overexpression of miR-30e arrested cell proliferation, but this was rescued upon coexpression of exogenous IRS1 in MDA-MB-231 cells. (**b**) Cells were treated as above. A sterile 200 μl pipette tip was used to scratch the cells to form a wound. The wound gaps were photographed (top) and measured (bottom). Forced expression of miR-30e also markedly reduced the wound-healing rate, and overexpression of IRS1 reverses the inhibitory effects of miR-30e. (**c**) MiR-30e overexpression decreased cell invasion in MDA-MB-231 cells. Cells were transfected with miR-30e followed by IRS1 transfection. All cells were subjected to a Matrigel invasion assay. (**d**) MDA-MB-231 cells stably expressing miR-NC or miR-30e were pretreated with various concentration of paclitaxel for 48 h, and subjected to CCK8 Assay. (**e**,**f**) MDA-MB-231 cells stably expressing miR-NC, miR-30e or miR-30e forced expression of IRS1 were pretreated with 4 nM of paclitaxel for definite time points, and subjected to CCK8 Assay, apoptosis analysis by flow cytometry. (**g**) MDA-MB-231cells stably expressing miR-NC or miR-30e were transfected with 2 μg pCMV6 vector or pCMV6–IRS1 plasmid and cultured with or without paclitaxel. After 72 h, the relative caspase-3 activities were determined. Data represent mean ± SD. of 3 replicates. * or ^#^ indicated significant difference at *P* < 0.05. **Indicated significant difference at *P* < 0.01. *Indicates significant difference compared to control; ^#^indicates significant difference compared to miR-30e plus IRS1 treatment.

Given the important role of IRS1 in regulation of cell proliferation, cell migration and invasion, miR-30e-overexpressing MDA-MB-231 cells were used to analyze cell growth and migration. The results showed that cell growth and migration were attenuated in miR-30e-overexpressed MDA-MB-231 cells compared with MDA-MB-231 cells expressing miR-NC (Fig. 4a,b). To test the role of IRS1 in cellular function, we showed that forced expression of IRS1 restored miR-30e-inhibited cell proliferation and migration (Fig. 4a,b). Then, we investigated the effects of miR-30e on invasion *in vitro*. Over-expression of miR-30e dramatically inhibited the normally strong invasive capacity of MDA-MB-231 cells (Fig. 4c). Finally, our results suggested that overexpression of miR-30e suppressed cell proliferation, cell migration and invasion by inhibiting IRS1.

Resistance to paclitaxel treatment is one of the major causes for the failure of chemotherapy in treating breast cancer. Therefore, it is critical to increase the effectiveness of paclitaxel for therapeutic purposes. Our results showed that overexpression of miR-30e in breast cancer cells significantly increased chemosensitivity to treatment of paclitaxel (Fig. 4d). Furthermore, cell growth rate in the presence of paclitaxel(4 nM) was assayed by CCK-8 proliferation assay at different time points; interestingly, forced expression of IRS1 reversed miR-30e-induced breast cancer cell chemosentivity to paclitaxel (Fig. 4e). To further study whether miR-30e and its target IRS1 play a role in cell apoptosis in the presence of paclitaxel treatment, FACS analysis was performed to detect cell apoptosis rates. The combination of miR-30e and paclitaxel treatment significantly induced cell apoptosis, whereas forced expression of IRS1 partially abolished the effect induced by miR-30e plus paclitaxel treatment (Fig. 4f). We also found that the activity of caspase-3, a key executor of cell apoptosis, was significantly upregulated upon treatment by miR-30e plus paclitaxel compared with miR-30e or paclitaxel treatment alone, whereas IRS1 overexpression attenuated the activation of caspase-3 induced by miR-30e plus paclitaxel treatment (Fig. 4g). These results indicated that miR-30e renders breast cancer cells more sensitive to paclitaxel treatment, miR-30e and paclitaxel combination induced apoptotic effect through targeting IRS1 in breast cancer cells.

### MiR-30e inhibits tumor growth *in vivo*

**Figure 5:**
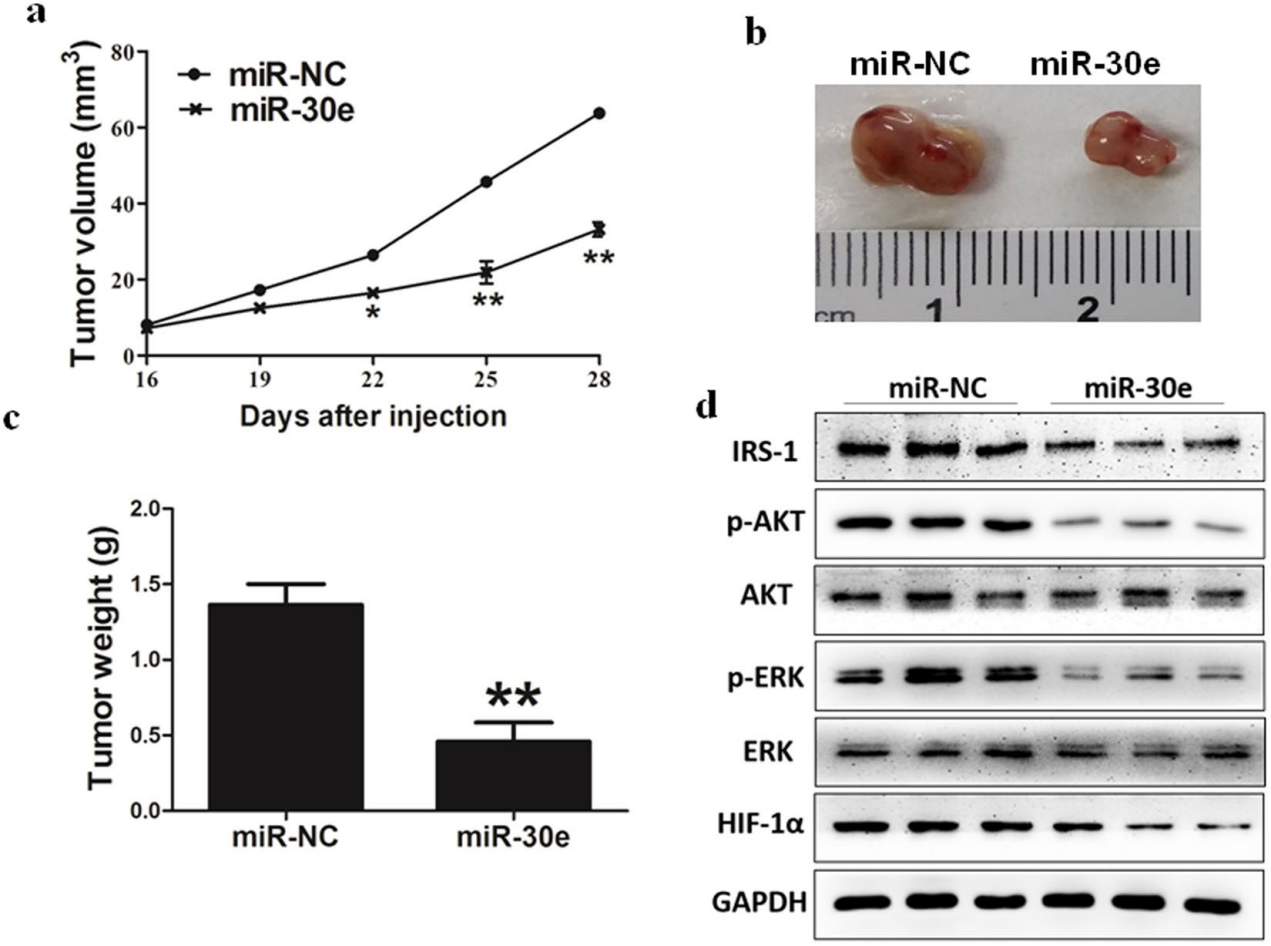
MiR-30e inhibits tumor growth *in vivo*. (**a**–**c**) Effect of miR-30e on the growth of MDA-MB-231 cells inoculated into nude mice. Male BALB/cA nude mice were subcutaneously injected with 5 × 10^6^ MDA-MB-231 cells infected with lentiviruses harboring miR-NC or miR-30e. Tumor volume and weight were monitored over time as indicated, and the tumor was excised and weighed after 24 days. MiR-30e caused a decrease in tumor volume and weight. (**d**) The expression levels of IRS1 and downstream pathway proteins from the tumor tissues of miR-30e expressing group were lower than those of miR-NC group. Data represent mean ± SD. *Indicates significant difference at *P* < 0.05; **indicates significant difference at *P* < 0.01.

In order to investigate whether overexpression of miR-30e attenuates progression of BC *in vivo*, we conducted MDA-MB-231 cells to stably express miR-NC or miR-30e, then cells were subsequently implanted into both posterior flanks of immunodeficient mice and the tumor sizes were measured after 10 days. From the 2^nd^ to 4^th^ week, miR-NC-injected group developed significantly larger tumors than miR-30e group (Fig. 5a). MiR-30e-expressed cells generated xenografts that were statistically significantly smaller than control (Fig. 5b). Meanwhile, the final tumor weight of miR-NC group was much heavier than miR-30e group (Fig. 5c). In agreement with *in vitro* studies, the levels of IRS1 from the tumor tissues of miR-30e-expressing group were lower than that of miR-NC group by immunoblotting assay (Fig. 5d). Moreover, some downstream pathway proteins, such as p-AKT, p-ERK1/2 and HIF-1α were significantly suppressed by miR-30e in tissues (Fig. 5d). Consistent with our previous studies, these results suggested that miR-30e inhibited tumor growth through targeting IRS1 and other downstream signaling molecules *in vivo*.

## Discussion

In this study, we provided a novel molecular insight of miR-30e impacting BC by suppressing IRS1 expression. Recent studies have demonstrated that miRNAs play important roles in carcinogenesis, and certain miRNAs have been studied to be correlated with clinical characteristics and outcomes. The role of some miRNAs in BC has also been reported. For example, Mutlu M *et al*. found that miR-564 acts as a dual inhibitor of PI3K and MAPK signaling networks and inhibits proliferation and invasion in breast cancer^26^. Yu J *et al*. reported that microRNA-320a inhibits breast cancer metastasis by targeting metadherin^27^. Lian JB *et al*. found that antagonizing miR-218-5p attenuates Wnt signaling and reduces metastatic bone disease of triple negative breast cancer cells^28^. Zhu J *et al*. found that Downregulation of microRNA-27b-3p enhances tamoxifen resistance in breast cancer by increasing NR5A2 and CREB1 expression^29^. Furthermore, increasing evidence indicates that miRNAs are playing a role in BC progression by regulating cell proliferation, cell migration, cell invasion and chemoresistance.

In the present study, miR-30e expression was significantly reduced in BC tissues and cell lines when compared with normal controls, respectively. Previous studies have shown that miR-30e is down-regulated in several cancer types. Feng *et al*. found that miR-30e suppresses proliferation of hepatoma cells via targeting prolyl 4-hydroxylase subunit alpha-1 (P4HA1) mRNA^21^. Granot G *et al*. confirmed that miR-30e induces apoptosis and sensitizes K562 cells to imatinib treatment via regulation of the BCR-ABL protein^22^, and miR-30e regulated Ubc9 expression in cancer cells^24^. Among others, miR-30e expression was upregulated in hepatoma patients who did not respond to cisplatin-therapy^30^. It is not surprising that miRNA can modulate different target expressions depending upon disease and cellular contest. In this study, the miR-30e expression was down-regulated in human BC, and over-expression of miR-30e inhibits the ability of cell proliferation, migration and invasion in BC cells.

IRS1 transmits signals from insulin/IGF receptor to activate PI3K/AKT and MAPK pathways, both of which are critical in mitogenesis and oncogenesis^31,32^. In our study, IRS1 oncogene was validated as the novel target of miR-30e. Firstly, luciferase reporter assay found that miR-30e directly recognized the 3′-UTR of IRS1 transcripts. Secondly, the IRS1 expression was significantly abolished in BC cells expressing miR-30e. Finally, miR-30e suppressed constitutive phosphorylation of AKT and ERK1/2, and inhibited expression of HIF-1α and VEGF via targeting IRS1. Taken together, our study provided the first evidence that miR-30e played a significant role in suppressing BC cell growth through inhibition of IRS1. Although we confirmed that miR-30e could inhibit the phenotype of BC by targeting IRS1, there might be other targets of miR-30e, which could also affect the growth of BC cells. Therefore, future studies are required to identify additional targets and pathways of miR-30e.

Taken together, our study indicated that IRS1 was a direct downstream target of miR-30e and was involved in the miR-30e-induced suppression of the activity of cell migration and invasion in breast cancer cell. The miR-30e/IRS1 link may play a role in breast cancer as markers of metastasis and prognostic factors. Furthermore, the therapeutic value of miR-30e in reducing cancer invasion, metastasis and chemoresistance should be further validated by independent cohorts and prospective trials.

## Materials and Methods

### Clinical specimens

Human breast cancer specimens (30 pairs) and adjacent normal tissues were obtained from Nanjing Medical University and Department of Breast and Thyroid Surgery, Huai’an First People’s Hospital. All tissue samples were snap-frozen in liquid nitrogen immediately after surgery and stored in liquid nitrogen. All samples were histologically classified by clinical pathologist. Samples used for molecular analysis were initially grinded into powder in liquid nitrogen and then collected separately for RNA analyses. This study was approved by the institutional review board and the ethics committee of Nanjing Medical University. Informed consents were obtained from all subjects participating in the study.

### Cell culture and reagents

Human embryonic kidney cell line 293 T (HEK293T), Breast cancer cell lines (MCF-7, MDA-MB-231), and mammary epithelia cell (MCF10A), were obtained from American Type Culture Collection. Cells were incubated at 37 °C in a humidified chamber supplemented with 5% CO2. Antibodies against IRS1, p-AKT, AKT, p-ERK1/2 and ERK1/2 were purchased from Cell Signaling Technology (Danvers, MA, USA). Antibody against GAPDH was from Bioworld Technology (Atlanta, Georgia 30305, USA). The growth factor reduced Matrigel was from BD Biosciences (Bedford, MA, USA).

### Lentiviral packaging and stable cell line establishment

To stably overexpress miR-30e in breast cancer cells, the lentiviral packaging kit was used (Thermo Fisher Scientific). Lentivirus carrying miR-30e or negative control (miR-NC) was packaged using HEK293T cells following the manufacturer’s manual. The lentiviral vector has red fluorescent protein (RFP) tag which can be used to check the efficiency of packaging using microscope. Cells were infected by lentivirus carrying miR-30e or miR-NC in the presence of polybrene (Sigma-Aldrich) and selected by puromycin (Sigma-Aldrich) for two week to obtain stable cell lines.

### Total RNA extraction, reverse transcription PCR and quantitative real time-PCR

Total RNAs were isolated from harvested cells or human tissues using Trizol reagent according to the manufacturer’s instruction (Invitrogen, CA, USA). To measure the expression levels of miR-30e, qPCR assay was performed and the U6 was used as an endogenous control. To determine the mRNA levels of IRS1, total RNAs were reversely transcribed by oligodT primer using PrimeScript RT Reagent Kit (Takara, Dalian, China). Housekeeping gene GAPDH was used as internal control. The cDNAs were amplified by real-time PCR using SYBR Premix DimerEraser (Takara, Dalian, China) on a 7900HT system, and fold changes were calculated by relative quantification (2^−△△Ct^).

### Immunoblotting

Cells were washed with ice-cold PBS buffer, scraped from the dishes, and centrifuged at 12,000 rpm, 4 °C for 15 min. Cell lysates were prepared using RIPA buffer supplemented with protease inhibitors (100 mM Tris, pH 7.4, 150 mM NaCl, 5 mM EDTA, 1% Triton X-100, 1% deoxycholate acid, 0.1% SDS, 2 mM phenylmethylsulfonyl fluoride, 1 mM sodium orthovanadate, 2 mM DTT, 2 mM leupeptin, 2 mM pepstatin).The supernatants were collected and protein concentrations were determined using BCA assay (Beyotime Institute of Biotechnology, Jiangsu, China). Tumor tissues from human and nude mice were grinded into powder in liquid nitrogen with RIPA buffer, and the total tissue proteins were extracted as described above. Aliquots of protein lysates were fractionated by SDS-PAGE, transferred to a PVDF membrane (Roche, Switzerland), and subjected to immunoblotting analysis according to the manufacturer’s instruction. ECL Detection System (Thermo Scientific, Rockford, IL, USA) was used for protein signal detection.

### Cell proliferation assay

To evaluate the proliferation effect of miR-30e in breast cancer cells, stable cell lines were plated at a density of 2 × 10^3^ per well in 96-well plate incubated at 37 °C in 5% CO_2_ incubator. The cell proliferation was measured using a CCK-8 kit (Dojindo Laboratories, Kumamoto, Japan) according to the manufacturer’s instruction. Data were from three separate experiments with six replications per experiment.

### Wound healing assay

Cells were transfected with miR-30e or miR-NC according to the manufacturer’s instructions, and then cells were cultured to 95% confluence in 6-well plates. Cell monolayers were scratched using a 20 µL tip to form wound gaps and washed twice with PBS to remove the detached cells. After 24 h, the wound healing was photographed at different time points. The cell migration distances were measured in three different areas to indicate the migration ability of different treated cells.

### *In vitro* Invasion assay

Invasion assay was determined using 24-well BD Matrigel invasion chambers (BD Biosciences, Cowley, UK) in accordance with the manufacturer’s instruction. Indicated cells were plated at a density of 5 × 10^4^ per well in the upper chamber without serum. The lower chamber was filled with 600 μl of the DMEM medium with 10% FBS to act as the nutritional attraction. After incubation for 24 h, noninvading cells were removed from the top well with a cotton swab, while the bottom cells were fixed with 3% paraformaldehyde, stained with 0.1% crystal violet, and photographed in three independent fields for each well. They were finally extracted with 33% acetic acid and detected quantitatively using a standard microplate reader (OD at 570 nm). Three independent experiments were conducted in triplicate.

### Dual-luciferase reporter assay

For dual-luciferase assay, 3′-UTRs of IRS1 containing predicted miR-30e seed-matching sites and corresponding mutant sites were amplified by PCR using human cDNA template, and inserted into the SacI and HindIII restriction enzyme sites by pMIR-REPORTER vector (Ambion, CA, USA). These constructs were validated by the DNA sequencing. Cells were seeded in a 24-well plate and co-transfected with the wild type or mutant reporter plasmid, pRL-TK plasmids, and miR-30e or miR-NC. Luciferase activities were analyzed 24 h after transfection using the Dual Luciferase Reporter Assay System (Promega, WI, USA). Experiments were performed in three independent replicates.

### Chemosensitivity array

Cancer cells were seeded at a density of 4,000 cells per well in a 96-well plate. 24 h later, freshly prepared paclitaxel (Sigma-Aldrich, St. Louis, MO, USA) was added with the final concentration ranging from 1 to 32 nM. 48 h later, cell viability was assayed by CCK8 kit.

### Flow cytometry assay

Apoptosis were measured by flow cytometry. For AnnexinV staining, 5 μL phycoerythrin-Annexin V, 5 μL propidium iodide (BD Pharmingen) and 300 μL 1 × binding buffer were added to the samples, which were incubated for 15 min at room temperature in the dark. Then the samples were analyzed by flow cytometry (FACS Canto II, BD Biosciences) within 1 h. The data were analyzed using FlowJo software. Three experiments were performed in triplicate.

### Caspase-3 Activity Assay

The activity of caspase-3 was determined using the Beyotime caspase-3 activity kit. Cell lysates were prepared and incubated with reaction buffer containing caspase-3 substrate (Ac-DEVDpNA) after the treatment as indicated. Caspase-3 activity assay were performed on 96-well plates by incubating 10 μL protein of cell lysate per sample in 80 μL reaction buffer containing 10 μL caspase-3 substrate(Ac-DEVD-pNA; 2 mM) at 37 °C for 2 h according to the manufacturer’s protocol. The reaction was then measured at 305 nm for absorbance.

### *In vivo* tumorgenesis assay

Male nude mice (BALB/c-null, 6-week-old) were purchased from Shanghai Laboratory Animal Center (Chinese Academy of Sciences, Shanghai, China), and bred in special pathogen-free (SPF) condition. Cells (5 × 10^6^) were suspended in 150 μl of serum-free DMEM medium, and injected subcutaneously into each side of the posterior flank of the same nude mouse. Tumor sizes were measured every three days from the 14^th^ day. Tumor volumes were calculated using vernier caliper using the formula: volume = 0.5 × (Length × Width^2^). Mice were sacrificed 30 days after implantation, and tumors were dissected. Total proteins were extracted and used for immunoblotting. All animal experiments were approved by the Committee of Laboratory Animal Experimentation of Nanjing Medical University.

### Statistical analysis

All experiments were performed three times, and data were analyzed with GraphPad Prism 5(La Jolla, CA, USA). The correlations between miR-30e expression levels and IRS1 levels in human breast cancer tissues were analyzed using Spearman’s rank test. Statistical evaluation for data analysis was determined by *t*-test. The differences were considered to be statistically significant at *P* < 0.05.
